# Spermatozoa Obtained From Alpaca *vas deferens*. Effects of Seminal Plasma Added at Post-thawing

**DOI:** 10.3389/fvets.2021.611301

**Published:** 2021-02-10

**Authors:** Eduardo G. Aisen, Wilfredo Huanca López, Manuel G. Pérez Durand, Edita Torres Mamani, Juan C. Villanueva Mori, María J. Ousset, Víctor H. Medina, Uri H. Pérez Guerra, Teodosio Huanca Mamani

**Affiliations:** ^1^Laboratorio de Teriogenología “Dr. Héctor H. Morello,” Instituto de Biotecnología del Comahue-Centro de Investigaciones en Toxicología Ambiental y Agrobiotecnología del Comahue (Consejo Nacional de Investigaciones Científicas y Técnicas-Universidad Nacional del Comahue), Facultad Cs. Agrarias, Universidad Nacional del Comahue, Cinco Saltos, Argentina; ^2^Laboratorio de Reproducción Animal, Facultad de Medicina Veterinaria, Universidad Nacional Mayor de San Marcos, Lima, Peru; ^3^Laboratorio de Reproducción Animal y Laboratorio de Biotecnología de la Reproducción, Facultad de Medicina Veterinaria y Zootecnia, Universidad Nacional del Altiplano, Puno, Peru; ^4^Instituto Nacional de Innovación Agraria - Programa Nacional de Camélidos, Estación Experimental Quimsachata-ILLPA-Instituto Nacional de Innovación Agraria, Puno, Peru

**Keywords:** seminal plasma, alpaca spermatozoa, sperm cryopreservation, *vas deferens*, South American camelids

## Abstract

The viscous seminal plasma (SP) is currently a major impediment to the handling of ejaculate and the development of some biotechnologies in South American camelids. The *vas deferens*-collected spermatozoa of alpacas is a useful technique to avoid this problem. On the other hand, SP contains a large protein component that has been implicated in the function of spermatozoa within the female reproductive tract. In this sense, the low fertility achieved using transcervical insemination with frozen-thawed spermatozoa in alpacas could be improved by adding SP. This study aimed to evaluate the effect of the whole SP on some *in vitro* parameters of alpaca spermatozoa after the freezing-thawing-process and the fertility after artificial insemination. It would contribute to a better understanding of the interaction between thawed sperm cells and SP. Spermatozoa were obtained by surgically diverted *vas deferens*. The samples were diluted with a Tris-based extender, packaged in straws, and frozen. At thawing, each straw was divided into two post-thawing conditions: with the addition of 10% of PBS (control) or with 10% SP (treatment). The sperm cells were evaluated using dynamic parameters, sperm cell morphology, and morphometry. Fertility was assessed by an artificial insemination trial. All *in vitro* parameters were analyzed by ANOVA. A heterogeneity test was scheduled for the fertility trial. After the freezing-thawing process, motility and plasma membrane functionality was improved when SP was added. No differences were found for post-thaw viability between the control and treatment samples. The percentage of normal cells was higher with SP at post-thawing, and a decrease of the presence of bent tailed spermatozoa with a droplet in the SP group was observed. The length of the head spermatozoa was 3.4% higher in the samples with PBS compared to those in which SP was added. Females pregnant at day 25 post-insemination were 0/12 (with SP inside the straw) and 1/10 (without SP inside the straw). In conclusion, the presence of 10% SP at post-thawing improves sperm cells' motility, functionality, and morphology, indicating that it would be beneficial to improve the frozen-thawed alpaca's physiology spermatozoa. More fertility trials must be developed to increase this knowledge.

## Introduction

The most remarkable characteristics of semen in South American camelids are its high structural viscosity and thread formation (1–3). These rheological characteristics impede the homogeneous mixing of semen with the extender, thereby limiting contact between the sperm cell membrane and cryoprotective compounds during the cooling and freezing processes (4–6), consequently hindering the development of artificial insemination technologies for this species (7).

To reduce the thread formation, semen from llamas was incubated in a 0.1% collagenase solution (1). Working with Bactrian camel semen, the ejaculates were treated with a magnetic stirrer at low speed (100 rpm) for 5 min (8), providing a liquefied homogenous specimen. Recently, the combination of the slow mechanical method (aspirating and expelling with a syringe or pipette) with low gravity acceleration (700 × g) allows obtaining SP without sperm cells [(9) for llamas; (10) for alpacas], avoiding the possible expelling of fluids (including proteins) from the damaged spermatozoa.

To avoid the problem of high viscosity, several authors decided to develop their experiments using epididymal or *vas deferens*-collected spermatozoa (11, 12). Pérez et al. (13) demonstrated that it is possible to obtain sperm cells from surgically diverted *vas deferens* in male alpacas and llamas, thus facilitating the evaluation of concentration, motility, abnormalities, and subsequent cryopreservation. This kind of collection may be useful for research purposes.

However, SP contains a large protein component that has been implicated in the function, transit, and survival of spermatozoa within the female reproductive tract (14). These authors identified a total of 10 alpaca SP proteins. Those that are in the 10–25 kDa range have an important modulation effect on sperm functionality. For example, RSVP14, a binder of sperm protein that can protect ram sperm membranes from cold shock (15), is present in alpaca SP, suggesting a common role of this seminal plasma protein on sperm functions in various species. In llamas, it was observed that sperm bind to N-acetylgalactosamine (GalNAc) on the surface of the oviductal epithelium. This condition is necessary to establish the oviductal sperm reservoir of South American camelids (16). More recently, seminal lectin SL15 was studied. This lectin is likely presented to sperm via seminal plasma since epididymal sperm cannot bind GalNAc, whereas ejaculated sperm does (17). These findings have great importance in explaining SP's relevance of the functions of ejaculated sperm and sperm cells obtained from epididymis or *vas deferens*.

Alpacas, like other Old and New World camelids, are classified as an induced ovulating species. They need external stimulation during the copulation to develop ovulation (18). Alpaca and camel SP contains a 13 kDa protein identified as beta nerve growth factor (β-NGF), which plays an important role in ovulation in this species (3). Although the effect of β-NGF on South American camelids sperm motility has not been studied (6), the co-location of β-NGF with tyrosine kinase receptor A (TrKA) in the middle piece of ejaculated and acrosome reacted llama sperm (19), suggest a possible action on sperm motility.

With several compounds, SP can both inhibit and stimulate sperm function and fertility through multifunctional actions (20). It is well known that some proteins of SP linked to spermatozoa are necessary to achieve fertility and oocyte binding. They were identified in alpaca SP and are similar to those reported in other species (5). The addition of SP to sperm, following cryopreservation, increased post-thawed motility and fertility in ram (21), enhanced post-thawed sperm function in boar (22), and increased artificial insemination fertility in stallion (23). In alpaca, it was demonstrated that 10% of SP incubated with sperm cells recovered from the epididymis preserve motility, acrosome, and viability of spermatozoa (24). In contrast, when SP was added to thawed llama semen, it was incapable of preserving motility or membrane function during 3 h of incubation (6). It was observed that SP from alpaca males with high semen freezability are related to specific protein fractions (14–15 kDa), which are absent in SP from males with low semen freezability (25). These findings allow us to conclude that SP is necessary to maintain the survival and fertilizing capacity of spermatozoa in camelids.

Furthermore, few trials use transcervical insemination with frozen-thawed spermatozoa in alpacas, and the fertility achieved is null or very low (26). Explanations for this low fertility include several factors such as the time between insemination and ovulation, the number of sperm cells, the volume of the dose, handling of the frozen-thawed semen, use of females with doubtful fertility, and others (27).

This study aimed to evaluate the effect of whole SP on some *in vitro* parameters of alpaca sperm cells after the freezing-thawing-process and the fertility rate achieved after an artificial insemination trial.

## Materials and Methods

### Animals

Five adult alpaca males (>4 years old with proven fertility) were used to obtain sperm cells from *vas deferens*. Animal welfare conditions were ensured in accordance with institutional statements (approved by Ethics Committee, Facultad de Veterinaria y Zootecnia, Universidad Nacional del Altiplano, Puno, Perú, 15° 49′ 34.5″ S, 70° 00′43.5″ W, 3 820 MSL). Spermatozoa were obtained by pipette aspiration of surgically diverted *vas deferens*. This technique is described by Pérez et al. (11, 13) and consists of five steps: (a) the males were fasted for 24 h before the surgery and were chemically restrained with Acepromazine maleate (0.1 mg/Kg BW); (b) the animals were placed in a supine position, and the surgical field was prepared in the inguinal region to perform local anesthesia; (c) a small incision (4 cm) was made in the skin over the penis; (d) the *vas deferens* of the right and left side were located and dissected in a length of 7 cm; (e) then, the dissected parts of *vas deferens* were redirected below the subcutaneous tissue and fixed to the skin of the internal face of the femoral region, and protected with a temporary patch.

### Reagents

All chemical reagents employed were of the highest commercially available purity, purchased from Sigma–Aldrich Co. (Saint Louis, USA).

### Sperm Cells Recovery and Process

Before each recovery, the fistula's opening and the skin near this hole were washed with sterile PBS. The recovery process was begun with a rectilinear massage of the *vas deferens* with the fingertips, allowing the sperm cells to move toward the outflow of the fistula. As the drops with spermatozoa appeared, they were aspirated by means of an automatic pipette with a tip moistened in a Tris-based extender. The drops (about 80–100 μL) containing the sperm cells were quickly diluted with a Tris-based solution and placed in a plastic tube at 37°C. The samples were assessed through total sperm cells (TS), sperm cell concentration, sperm motility (TM), and sperm cell morphometry (28). Only samples with TS higher than 20 × 10^6^ spermatozoa and TM higher than 50% were used.

A total of 20 samples (five males × four repetitions) were performed. The collected and evaluated samples were diluted with Tris (240 mM), citric acid (76 mM), glucose (22 mM), glycerol (3% v/v), and egg yolk (10% v/v) to a final concentration of 50 × 10^6^ spermatozoa/mL (29). After the dilution and cooling processes, the mixtures were stabilized at 5°C for 90 min. They were then packaged in 0.25-mL straws (IMV Technologies, L'Aigle, France) and frozen in liquid nitrogen vapor (−100°C). For the freezing process, the straws were placed on a rack inside a Styrofoam box. The height was graduated manually, remaining 10 min at 24 cm, 10 min at 12 cm, and 5 min at 3 cm above the liquid nitrogen level. Finally, the straws were plunged into the liquid nitrogen and stored in a nitrogen tank at −196°C [modified from (30)].

### Sperm Cells Treatments

Thawing was performed by immersion in a water bath for 20 s at 37°C. Each straw was divided into two post-thawing conditions: with the addition of 10% PBS (control) or with 10% SP (treatment). Both fractions were incubated at 37°C for 20 min. Seminal plasma was obtained from ejaculates of six adult alpaca males, during the breeding season, under the same animal welfare conditions indicated previously. For this purpose, semen samples obtained by means of an artificial vagina were treated using a slow mechanical method (9, 31) to reduce thread formation and to obtain a more fluent sample. Briefly, each ejaculate was very slowly aspirated and expelled (avoiding foam formation) with a needle (0.5 mm inner diameter) attached to a syringe, repeating this action 10 times. Then, the ejaculates were centrifuged at 700 × g for 30 min (10), and the supernatant was recovered and centrifuged once again. SP was then recovered, and a drop was evaluated by microscopy to confirm the absence of cells (32). The SP obtained was polled and stored at −20°C before use.

### Polyacrylamide Gel Electrophoresis (SDS-PAGE)

An electrophoresis was carried out to obtain an approximation of the molecular weight of the proteins present in the SP prepared for this experiment. Six sources (males) of SP were electrophoresed in 12% polyacrylamide gels containing sodium dodecyl sulfate (SDS-PAGE) at room temperature (Mini Protean 3-Bio-Rad Laboratories, Inc, California, USA), as described by Laemmli (33). The total amount of 11 μg of protein, quantified by the Bradford (34) method, was seeded in each lane. Molecular weight was estimated using protein molecular weight standards (PB-L Productos Bio-logicos, Buenos Aires, Argentina). The gels were stained with Coomassie blue and then washed, scanned (transmission acquisition), and observed.

### *In vitro* Assessment

After thawing and incubation with PBS or SP, the samples were evaluated *in vitro* using an inverted microscope (Nikon Eclipse Ti-S, Tokyo, Japan).

Sperm motility was determined by placing 10 μL of a sperm sample on a warm slide and covering it with a warm coverslip, using a warm stage (37°C) phase-contrast optics and video-microscopy (35).

Sperm motility index (ISM) was calculated as TM × movement quality (36). In this way, the quantity (percentage of motility) and quality (vigor) of sperm movement could be considered in a single parameter.

Plasma membrane functional integrity was assessed by the hypoosmotic swelling test (HOST), using a modified method developed by Giuliano et al. (37). This method consists of the incubation (37°C, 20 min) of 50 μL of sperm samples in 200 μL of hypoosmotic solution (2.45 mg/mL of fructose and 4.5 mg/mL of sodium citrate, adjusted to 50 mOsm). The incubation was stopped with a hypoosmotic formol solution, and then 200 spermatozoa were evaluated.

Cell viability was determined with eosin-nigrosin staining (Eo-Ni), according to Bloom (38) and Aisen et al. (28). On a warm stage, 10 μL of the sperm sample and 10 μL of the supravital staining solution (5 g/L of eosin Y, 100 g/L of nigrosin, 29 g/L of sodium citrate) were mixed. After 30 s, the mixed drop was smeared by sliding a coverslip in front of it, dried on air, and observed by bright field microscopy (400 x).

Sperm cell morphology was performed using the slides stained with eosin-nigrosin and Barth and Oko (39) classification, modified by Medina et al. (40).

Sperm cell morphometry was developed as described by Aisen et al. (28). A high-resolution color digital camera (DS-Ri-U2, Nikon, Tokyo, Japan) and software for processing microscopic images (Nikon NIS Elements Advanced Research) were used. The head length (L-head) and head width (W-head) were measured, and ellipticity (E) was calculated, such as the L-head/W-head ratio.

### Fertility Trial

**Figure 1:**
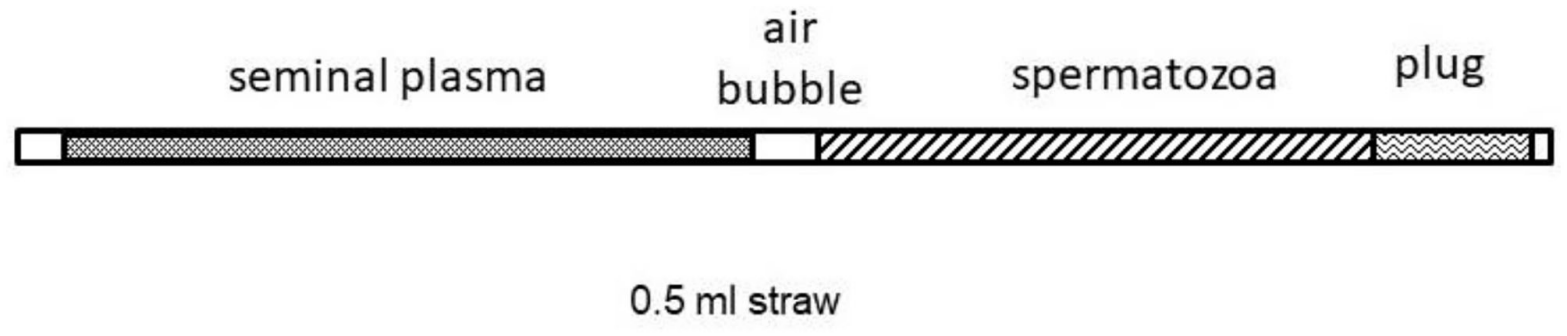
Scheme of straw loading with seminal plasma and spermatozoa.

Fertility was assessed by an artificial insemination trial (extensive field conditions). Spermatozoa from *vas deferens* (four males) were frozen into 0.5 mL-straws containing 20 × 10^6^ total sperm cells in two different volumes. One batch was loaded only with sperm cells + diluent (0.4 mL). The other batch (SP treatment) was filled partially with SP (0.2 mL) and loaded behind an air bubble to separate the spermatozoa + extender (0.2 mL); those were loaded at the end (Figure 1).

Each straw was separately thawed in a water bath (37°C) for 20 s. They were managed carefully, especially those containing SP to keep the two parts separate. The straws were dried with absorbent paper and placed into a universal semen applicator (IMV Technologies, France). Finally, this AI device was protected with a sterile sheath before seeding the female tract.

A group of adult alpaca females (Huacaya breed) from the Quimsachata Research Station (ILLPA-INIA, Puno, Perú, 15°45′ S, 70°34′ W, 4200 MSL) were destined to induce ovulation as described by Adams et al. (32). Considering that South American camelids are species of induced ovulation, the animals were subjected to the following maneuvers. Alpaca females were selected according to their ovarian dynamics. For this purpose, the presence of a dominant follicle ≥ 7 mm, detected by transrectal ultrasonography (Esaote portable ultrasound, 5 MHz probe, Genoa, Italy), was the condition to include them in the test. After performing the ultrasound examination, ovulation was induced with 1 ml of SP given intramuscularly (32). For this purpose, semen from adult alpaca males, obtained with an artificial vagina, was centrifuged at 1,500 × g for 15 min, and the supernatant was observed for the absence of sperm cells. A 1:1 SP dilution with PBS + antibiotics (kanamycin sulfate 25 μg/ml) was stored at −18°C until the moment of ovarian stimulation.

Two groups (12 and 10 females, with or without SP into the straws, respectively) were inseminated around 26 h after seminal plasma stimulation. The animals were restrained in stocks, the rectum was emptied of feces, and the perineum was cleaned. A lubricated, gloved hand was placed in the rectum, and the anatomical structures were located (cervix and bifurcation of the uterine horns). With the other hand, the vulva labia were separated, and the AI device was introduced into the vagina at an angle of approximately 40° upwards. With the index finger guiding the semen applicator, the cervix was threaded and traversed. The device was then directed to the uterine horn corresponding to the side where an ovarian with a dominant follicle ≥ 7 mm was observed (7). The seeding of the straw's content was performed as deep as possible in the uterine horn (closest to the uterotubal junction), slowly depressing the injector plunger.

Pregnancy was diagnosed by ultrasonography on Day 25 post-insemination, using the same ultrasound equipment, to locate an anechoic structure in the uterus, compatible with the embryonic vesicle (41).

### Statistical Analyses

All *in vitro* parameters were analyzed by ANOVA (main effects), with a Fisher–LSD *post hoc* test, using the software StatSoft, Inc. (2007). A heterogeneity test was planned for the fertility trial.

## Results

### Assessment of Spermatozoa Obtained

The values of raw sperm cell samples obtained from *vas deferens*, expressed as means ± standard error of the mean, were TS = 35.53±8.44 × 10^6^ spermatozoa; sperm concentration = 405.00 ±107.54 × 10^6^ cells/mL; TM = 57.50 ± 6.86 %; L-head = 6.06 ± 0.06 μm; W-head = 3.67 ± 0.03 μm; E = 1.65 ± 0.02. TS and TM were strongly affected by male factor.

### Identification of Molecular Weight of SP Proteins

**Figure 2:**
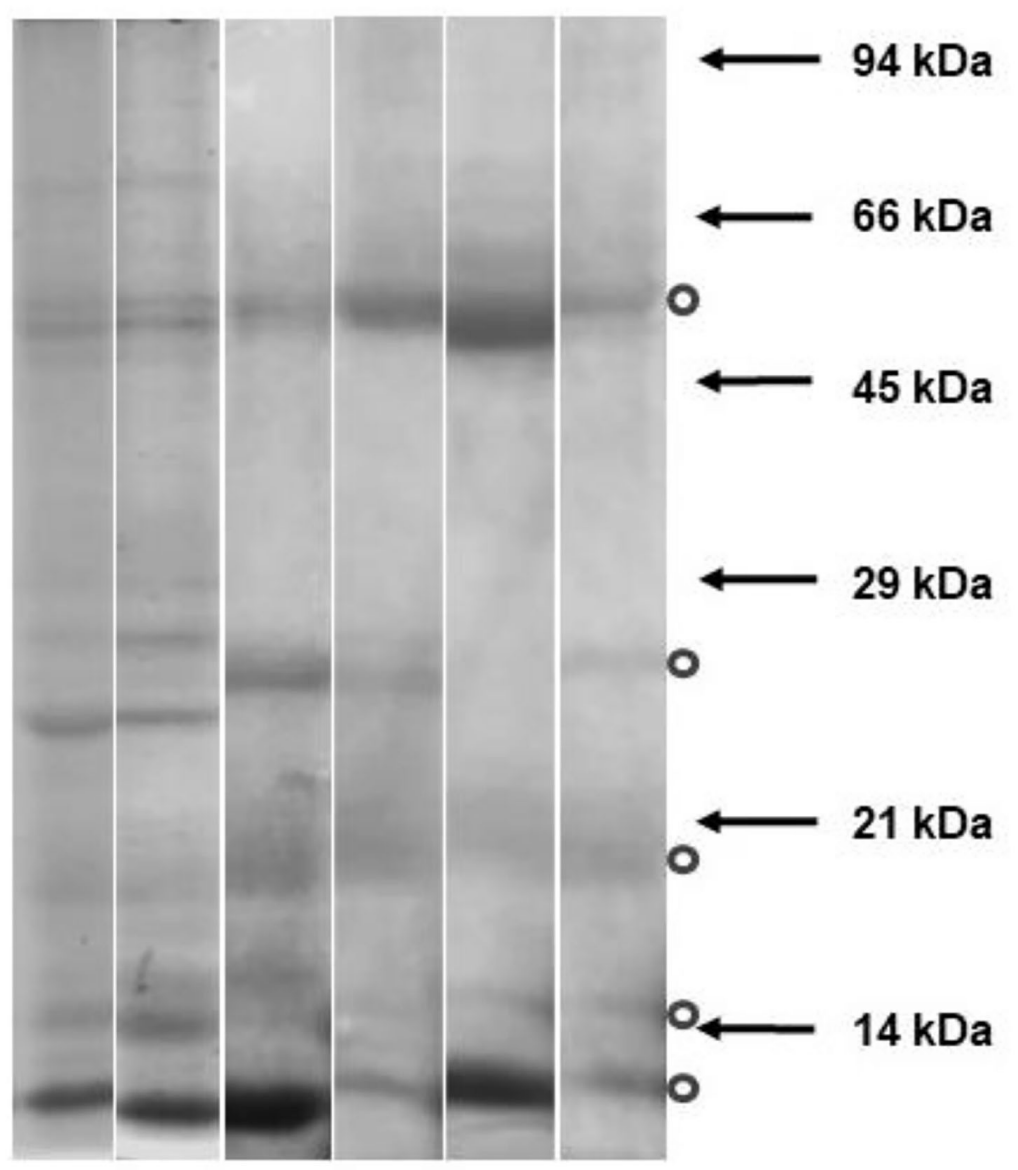
Protein bands observed by SDS PAGE of Seminal plasma from six alpaca males. Six seminal plasmas were electrophoresed in polyacrylamide gels containing sodium dodecyl sulfate (Mini Protean 3-Bio-Rad Laboratories, Inc, California, USA). Molecular weight was estimated using protein molecular weight standards (positions indicated with arrows). The gels were stained with Coomassie blue. Dots indicate most frequent bands observed.

The protein bands observed by SDS PAGE of SP are shown in Figure 2. Five single or group of bands were identified, with a molecular weight around 13, 14, 20, 25, and 60 kDa.

### *In vitro* Effects of SP at Thawing

At thawing, no interactions between males, dates, and replicates were found. The sperm cell dynamic parameters at thawing are shown in Table 1. The presence of SP after the freezing-thawing improved the TM (*p* = 0.035), ISM (*p* = 0.017), and HOST (*p* = 0.022), compared to the PBS treatment. No differences were found for Eo-Ni with respect to the addition of SP.

**Table 1:** Effect of seminal plasma added at post-thawing on frozen-thawed spermatozoa obtained from alpaca *vas deferens*. Sperm parameters (total motility, sperm motility index, viability, and membrane function).

**Treatment**	**TM** **(%)**	**ISM** **(%)**	**Eo-Ni** **(%)**	**HOST** **(%)**
With PBS	18.3 ± 2.1	40.0 ± 3.2	61.4 ± 2.5	41.5 ± 1.3
With SP	25.8 ± 2.42	78.5 ± 9.0	63.4 ± 1.4	48.2 ± 1.7
p value	0.035	0.017	0.510	0.022

Values are mean ± standard error.

*SP, seminal plasma; TM, total motility; ISM, sperm motility index; Eo-Ni, supravital stain; HOST, plasma membrane functionality. The parameters were analyzed by ANOVA (main effects), with Fisher–LSD post hoc test*.

The sperm cell morphology parameters are shown in Table 2. Normal sperm cell morphology was higher (*p* = 0.022) when SP was present at thawing, compared with PBS treatment (60.90 ± 1.40% vs. 54.60 ± 1.40%, respectively). A decrease in the presence of bent tailed with droplet spermatozoa was observed in the SP group with respect to the control group (0.45 ± 0.32% vs. 2.90 ± 1.03%, respectively).

**Table 2:** Effect of seminal plasma added at post-thawing on frozen-thawed spermatozoa obtained from alpaca *vas deferens*. Sperm morphology.

**Sperm morphology category**	**Treatment**		***p* value**
	**With PBS (%)**	**With SP (%)**	
Normal sperm cells	54.60 ± 1.42	60.90 ± 1.40	0.022
Small head	19.45 ± 1.25	18.79 ± 1.65	0.754
Detached normal head	1.53 ± 0.59	2.22 ± 0.62	0.347
Detached abnormal head	0.13 ± 0.02	0.45 ± 0.02	0.229
Abaxial head	2.10 ± 0.59	1.27 ± 0.69	0.389
Bent tail with droplet	2.90 ± 1.03	0.45 ± 0.32	0.04
Bent tail	12.30 ± 1.88	11.53 ± 0.93	0.721
Coiled tail	1.25 ± 0.61	1.33 ± 0.61	0.91
Proximal droplet	3.91 ± 1.61	1.69 ± 0.81	0.247
Distal droplet	1.44 ± 0.91	0.56 ± 0.36	0.393
Fractured neck	0.80 ± 0.60	0.68 ± 0.54	0.787

Values are mean ± standard error.

*SP, seminal plasma. The parameters were analyzed by ANOVA (main effects), with Fisher–LSD post hoc test*.

The sperm head cell morphometry parameters are shown in Table 3. The L-head showed a highly significant increase of about 3.4% (*p* = 0.0001) in the control samples (with PBS) compared to those where SP was added at thawing (6.29 ± 0.03 μm vs. 6.08 ± 0.03 μm, respectively). W-head and E were both 2% higher in the control samples with respect to those with SP added, showing statistical differences (*p* < 0.05).

**Table 3:** Effect of seminal plasma added at post-thawing on frozen-thawed spermatozoa obtained from alpaca *vas deferens*. Sperm head morphometry.

**Treatment**	**Head sperm cell morphometry**		
	**Length (μm)**	**Width (μm)**	**Ellipticity**
With PBS	6.29 ± 0.03	3.70 ± 0.03	1.73 ± 0.01
With SP	6.08 ± 0.03	3.62 ± 0.02	1.70 ± 0.01
*p* value	0.0001	0.026	0.037

Values are mean ± standard error.

*SP, seminal plasma. The parameters were analyzed by ANOVA (main effects), with Fisher–LSD post hoc test*.

### *In vivo* Effects of SP at Thawing

The females who were pregnant at Day 25 post-insemination were 0/12 (with SP inside the straw) and 1/10 (without SP inside the straw). No significant differences were found between the groups (*p* = 0.26).

## Discussion

The pipette aspiration of surgically diverted *vas deferens* seems to be a useful technique to obtain sperm cells from male alpacas, especially for the study of the freezing-thawing process (42). In this experiment, a high TS variation between males was observed, going from 5 × 10^6^ to 152 × 10^6^ total sperm cells. For this reason, and because of a linked low volume recovery, several samples were discarded. Frequently, working with very small volumes at the time of collection makes it difficult to handle and quickly protect against thermal changes, triggering the spermatozoa's damage or death. Pérez et al. (13) reported that this collection could be performed twice a week, obtaining about 25 × 10^6^ total spermatozoa at each collection, which is close to the value obtained in our work (35.53 ± 8.44 × 10^6^ spermatozoa). Motility of the spermatozoa recovered (57.50 ± 6.86 %) was similar to that achieved by Kershaw-Young and Maxwell in 2011 (56.3 ± 2.80%) and other authors for epididymal alpaca spermatozoa [52.7 ± 3.3% by (12) and 56.8 ± 9.8% by (13)].

After the freezing-thawing process, TM and sperm morphology related to the sperm tail status were improved when SP was added after thawing. In this case, the recovery of frozen-thawed spermatozoa was better, indicating SP's direct effect on some aspects of sperm cell physiology. Regarding this finding, it could be connected with the high ISM achieved. Motility of ejaculated or epididymal spermatozoa obtained from male alpacas (raw state) was improved when SP (especially at 10%) was added during incubation at 37°C (24).

Although no significant differences were observed for Eo-Ni at post-thawing in this experiment, an increase in live ram spermatozoa has been reported when adding 20% of SP at thawing. This addition caused the best values in the sperm quality parameters studied (43). In alpaca, Kershaw-Young and Maxwell (24) observed that the viability of ejaculated sperm was better when incubated in 10 rather than 100% seminal plasma. The cause of this reduced viability is not known, but may be associated with increased osmotic stress and lower pH due to the higher proportion of SP in the sample with 100% SP, which could affect the sperm parameters. Fumuso et al. (6) observed that the percentages of total live sperm were preserved over 3 h of incubation in all SP final concentrations evaluated (0, 10, or 50%), and no significant differences were observed in total live spermatozoa between the SP concentrations assayed. Although there is a coincidence with what was observed in our study (0 or 10% of SP), this work's results are not entirely comparable since the studies were developed using ejaculated spermatozoa, which had already come into contact with SP.

Regarding the results in this experiment, higher preservation of the plasma membrane functionality was observed, shown by HOST, indicating a possible beneficial interaction between SP proteins and the plasmalemma of the sperm cell. There are a few references to studies researching SP's effect on HOST using spermatozoa collected from the *vas deferens*. Zea et al. (44), evaluating spermatozoa from alpaca, found no differences in the addition of SP at thawing for this parameter. However, the percentage of SP used was not indicated in this work.

A group of five common protein bands was identified by SDS PAGE. Those with a molecular weight of around 13 and 14 kDa would correspond to βNGF and RSVP14, respectively (14). The bands below 25 kDa correspond to several proteins with a modulation effect on sperm functionality. The 20 kDa proteins prevent cold shock sperm membrane damage and show seasonal differences in ram SP proteins (15). Some of these proteins specifically bound to the acrosomal region of the ram sperm surface (45), such as the Lactotransferrin, epididymal secretory protein E1, Synaptosomal-associated protein 29, and RSVP-20 present in this fraction. The 60 kDa band proteins were observed as the most abundant in all male alpacas, without diet influence (46). It was demonstrated that not all SP proteins bound specifically to the sperm surface and improved the thawed sperm cells. These interacting seminal plasma proteins are not sufficient to emulate the effects of complete SP regarding sperm functional parameters (43). When certain proteins were incubated with frozen-thawed ram spermatozoa, they partially repaired semen cryodamage, protecting both the sperm motility and the ultrastructure (45). These authors demonstrated that sperm membrane was improved in frozen-thawed sperm cells treated for 15 min with SP proteins. These proteins could be sufficient to reverse molecular signals of capacitation caused by freezing, perhaps acting through the inhibition of the signal transduction pathways of capacitation (43). Some of these proteins are spermadhesins (heparin-binding proteins). They are the most likely protein fraction that binds to phospholipids on the sperm membrane upon ejaculation, stabilizing the sperm membrane, and preventing capacitation (14). Centurion et al. (47) incubated fresh boar spermatozoa with heparin-binding spermadhesins and non-heparin-binding spermadhesins. They found that non-heparin-binding spermadhesins contributes to maintaining sperm with high viability, motility, and mitochondrial activity for at least 5 h. They conclude that both spermadhesins exert antagonistic effects on the functionality of highly diluted boar spermatozoa. However, recent studies on llama ejaculates did not find improvements when SP was added after the freezing-thawing (6). When the results obtained in llamas are compared with those observed in our work on alpacas, it is important to consider the difference in the source of spermatozoa. In llama, ejaculated sperm had previous contact with SP, suggesting (but not reliably verified) that, in this case, the bounded proteins of the SP present in the semen at the time of ejaculation have previously exercised some protection against the cryopreservation. In the case of alpaca spermatozoa analyzed in our experiment, the first contact with SP was at post-thawing incubation.

There is a hypothesis (biphasic effect): SP interaction with spermatozoa could be beneficial in the short term in normal reproductive physiology but could be detrimental in the long-term preservation condition (48). Considering Kershaw-Young and Maxwell's (24) findings, most of the spermatozoa parameters incubated with SP measured at 0.5, or 1 h (motility, intact acrosomes, viability) showed the same behavior, predicting the dynamics during the incubation. However, most of the values of the parameters studied showed a strong decrease after 1 h. On the other hand, as indicated previously, ram sperm cells treated with SP for 15 min were sufficient at improving progressive motility and other parameters (45). For these reasons, we have considered exposing the sperm cell to the SP for a short time.

The morphology of frozen-thawed spermatozoa showed that the percentage of normal sperm cells was higher with SP at post-thawing. The principal differences were the values of the bent tailed with droplet cells (a secondary abnormality), indicating possible damage on tail plasma membrane permeability when the spermatozoa were incubated with PBS. Interestingly, the percentage of bent tails with droplets was close to zero when SP was present at post-thawing.

Continuing with sperm measurements, L-head was 3.4% higher in the control samples (with PBS) with respect to thawed samples with SP added. The analysis of SP's addition at post-thawing on the head morphometry allows supposing that, in this condition, SP modules the membrane permeability, slightly reducing the flow of water into the sperm cells because of the presence of glycerol at thawing (49). Due to the small capacity of the head sperm cell to change its volume, length may be the best parameter related to this modification compared to width. In ram spermatozoa, the ultrastructures below the plasma membrane (cytoskeleton included) seem to be less rigid at the acrosomal region than the equatorial segment or the post-acrosomal region (50, 51). It is also important to highlight that sperm cell morphometry parameters with SP at post-thawing (Table 3) were similar to those at the pre-freezing stage (L-head = 6.06 ± 0.06 μm; W-head = 3.67 ± 0.03 μm; E = 1.65 ± 0.02), allowing the cell to maintain the best physiological condition.

For the artificial insemination trial, the straws corresponding to the treatment group were filled with 0.2 mL of SP and 0.2 mL of sperm + extender. This original 1:1 ratio in the straw would not be maintained during seeding. It was considered that, during insemination, the part of SP (which is expelled at first) would be deposited on the uterine mucosa, losing a part of it due to the dilution effect in the uterine fluids. The part of thawed sperm cells that was seeded later would be deposited on this portion of SP, reaching a real final SP/sperm ratio much <50%, emulating a little more to *in vitro* conditions. Apichela et al. (52) confirmed that the presence of viscous mucus (in particular secreted by the bulbourethral glands) in the utero-tubal junction in llamas was involved in the formation of the sperm reservoir. This seminal factor can be modified when ejaculates are diluted with extenders, resulting in a diminished adhesion of sperm to the oviductal mucosa and the viability in the female tract. For this reason, SP added into the straw could artificially restore (partially) the conditions that sperm cells have after natural mating. This experimental design attempted to offer to thawed sperm (with a lower fertilizing capacity compared to fresh sperm) a more appropriate media to maintain their functions until reaching the oviduct. However, this condition could not be demonstrated during this fertility test.

This artificial insemination trial was carried out in field conditions, where it was not possible to perform tracing of the ovarian follicle's development. The follicular size was used as a guide to select those females that presented favorable conditions to induce ovulation. In this sense, Adams et al. (32) showed that, by selecting llamas with follicles larger than ≥8 mm, ovulation was detected in 90% of females after i.m. administration of seminal plasma. Unlike our work, females were previously induced to synchronize the follicular-wave emergence among animals with LH. More recently, Ascencio et al. (53) studied the effect of the stage of development of the follicular wave prior to the natural mating on the ovulation rate and the recovery rate and embryo quality in alpacas. These authors found that there were no significant differences between groups by size of the dominant follicle (phase of follicular growth, regression, or static), ovulation rate, or size of the corpus luteum on the day of embryo recovery. The lack of greater monitoring of ovarian dynamics in our work could have influenced the pregnancy rates obtained. Due to the low pregnancy values obtained, other factors such as the number of motile sperm cells and relocation of liquid components inside the straws should be considered in future experiments. The pregnancy diagnosis by ultrasonography was also performed at the 4th month, confirming that the only pregnant female remains in this state.

In conclusion, the presence of 10% SP at post-thawing improves sperm cell motility, plasma membrane functionality, and cell morphology, indicating that this condition would be beneficial to improve the physiology of the frozen-thawed alpaca spermatozoa. More *in vitro* and *in vivo* trials must be developed to increase knowledge of these findings.

## Data Availability Statement

The original contributions presented in the study are included in the article/supplementary material, further inquiries can be directed to the corresponding author/s.

## Ethics Statement

The animal study Plastic surgery of the vas deferens in South American camelids was previously reviewed and approved by the Institutional Animal Care and Use Ethics Committee of the Faculty of Veterinary Medicine and Zootechnics, Universidad Nacional del Altiplano (Perú), being applied to the present work.

## Author Contributions

EA: coordination, laboratory work and development of *in vitro* assays. WH: coordination and development of field trial. MP: development of surgically diverted vas deferens sperm recovery and development of *in vitro* assays. ET: surgically diverted vas deferens sperm recovery and laboratory work. JV: laboratory work and development of *in vitro* assays. MO: development of polyacrylamide gel electrophoresis (SDS-PAGE). VM: sperm morphology assessment. UP: laboratory work. TH: chief coordinator. All authors contributed to the article and approved the submitted version.

## Conflict of Interest

The authors declare that the research was conducted in the absence of any commercial or financial relationships that could be construed as a potential conflict of interest.
